# Comparison of antibacterial and antibiofilm activity of bioactive glass compounds S53P4 and 45S5

**DOI:** 10.1186/s12866-022-02617-8

**Published:** 2022-09-02

**Authors:** Peng Zhou, Brittny L. Garcia, Georgios A. Kotsakis

**Affiliations:** 1 Translational Periodontal Research Laboratory, UT Health San Antonio, San Antonio, TX USA; 2 Department of Periodontics, UT Health San Antonio, 7703 Floyd Curl Dr. 7894, TX 78229-3900 San Antonio, USA

**Keywords:** Bone substitutes, Biofilm, Ceramics / pharmacology, Bioactive glass, Anti-bacterial agents

## Abstract

**Background:**

Bone loss and deformation due to damage caused by injury or recurrent invasive infections presents a major clinical obstacle. While bone substitute biomaterials promote osseous tissue regeneration, their application in sites complicated by microbial infections such as osteomyelitis, is limited. Bioactive glass biomaterials (Bioglass) have been shown to have efficient mechanisms of repairing the integrity of bone, while inhibiting growth of a range of bacterial strains. There are several commercially available bioactive glass compounds, each with a unique chemical composition. One compound in particular, S53P4, has demonstrated antimicrobial effects in previous studies but the antimicrobial activity of the parent compound 45S5 has not been investigated.

**Results:**

To assess whether antimicrobial activity is common among bioglass compounds, 45S5-the parent compound, was evaluated in comparison to S53P4 for antibacterial and antibiofilm effects against multiple strains of aerobic and anaerobic bacteria associated with various types of osteomyelitis. Experiments of antimicrobial effects in liquid cultures demonstrated that both compounds were antimicrobial against various microbial genera including *S. gordonii*, *V. parvula*, *P. aeruginosa* and MRSA; particles of the smallest size (32–125 µm) invariably showed the most robust antimicrobial capabilities. When employed against biofilms ecological biofilms grown on hydroxyapatite, 45S5 particles produced a stronger reduction in biofilm mass compared to S53P4 particles when considering small particle ranges.

**Conclusion:**

We found that 45S5 seems to be as effective as S53P4 and possibly even more capable of limiting bacterial infections. The efficacy of bioactive glass was not limited to inhibition of planktonic growth, as it also extended to bacterial biofilms. The increased antibacterial activity of 45S5 compared to S53P4 is true for a variety of size ranges.

## Introduction

Globally, the need for bone and joint surgeries is constantly increasing, and bone substitute biomaterials are being developed to meet this demand as well as to optimize repair and regeneration of bone tissues [1]. While biomedical biomaterials research has mainly focused on tissue regenerative effects of bone substitute biomaterials, combating infections is equally as important as regenerating tissue. This need for both the ability to regenerate tissue and combat infection has shifted development focus towards multifunctional biomaterials with antibacterial properties. Bioglasses are bioactive materials which promote bone regeneration. Some bioglass formulations have been shown to have antibacterial properties, making them particularly suitable for use in compromised bone regeneration surgical procedures which have high infection risk, such as oral osseous and osteomyelitis defects [1]. The efficacy of S53P4 bioactive glass, a variant of 45S5, in inhibiting bacterial growth has been extensively documented both *in vitro* and *in vivo* [2–4]. Prior work has shown that S53P4 inhibits the growth of methicillin-resistant *Staphylococcus aureus* (MRSA), *Klebsiella pneumoniae*, and *Acinetobacter baumannii* by causing deformation of the cells, and hole formation in the cell membranes [5, 6]. In addition, bioglass 45S5 has been reported to exhibit an antibacterial effect against *S. aureus* and *Staphylococcus epidermidis* [7]. However, information is scarce on its antimicrobial effects due to a variety of size ranges. If equivalence can be determined, then the indications for 45S5 could be broadened to additionally include the indications for S53P4 related to clinical deployment in infected sites *in vivo,* as well as set the foundation for chemical optimization of antibacterial bioactive glass formulations.

Osteomyelitis itself is a costly medical issue; when antibiotic-resistant microbes are involved, it can lead to life-threatening complications. An array of bacteria has been implicated in both bone surgery complications and osteomyelitis resulting in substantial morbidity and medical costs. Usually, osteomyelitis is managed with IV antibiotic courses that may be empirical or adjusted based on microbial identification and requires several weeks of hospitalization [8]. One challenge is that the characteristics of the pathogens vary widely across clinical cases with an array of aerobic and anaerobic bacteria capable of destructive bone inflammation, thus limiting the efficacy of systemic antibiotics [9]. Furthermore, several pathogenic microorganisms that cause osteomyelitis are resistant to antibiotics. For instance, *Veillonella parvula*, which is a causative agent in vertebral osteomyelitis, was within the spectrum of Penicillin G and cephalosporins in the 1980s when its pathogenic role was established [10]. However, antibiotic targeting of *Veillonella* has led to considerable resistance in strains implicated in osteomyelitis and septicemia, with a recorded resistance to penicillin exceeding 30 μg/ml [11]. These findings highlight the need for developing local antimicrobial therapies for bone infections that are resistant to systemic antibiotic treatment.

In addition to preventing long-term resistance, it is essential to prevent systemic mortality and morbidity due to direct bacterial infection in bone lesions. In some cases, multidrug resistant bacteria implicated in osteomyelitis can lead to life-threatening complications. For instance, *Pseudomonas aeruginosa* is multidrug resistant, thus leading to mortality in hospitalized patients [12]. Similarly, MRSA is a notorious pathogen that causes severe infections in the bone. MRSA is more difficult to eliminate than most *S. aureus* strains due to its extraordinary capability of resistance to commonly used antimicrobials [13]. Additionally, *Streptococcus gordonii*, which is implicated in spondylodiskitis, can ultimately result in bacteremia and infective endocarditis [14, 15]. Therefore, the overall goal of this work is to identify whether the antimicrobial effects of multifunctional bioactive glass bone substitutes are present in all commonly employed compositions, and to determine efficacy against clinically relevant bacteria and their biofilms. The primary objective of this study is to compare anti-bacterial and anti-biofilm properties between Bioglass 45S5 and Bioglass S53P4 compositions.

## Materials and methods

### Bacterial strains and culture conditions

Representative Gram positive (Gram +) and Gram negative (Gram-) bacterial species / strains that have been found to be pathogenic in various forms of bone infections or osteomyelitis were used in this study; listed in Table 1. *S. gordonii* DL1 was grown in Brain Heart Infusion (BHI) broth (Oxoid™, Thermo Scientific™, USA), and *V. parvula* PK1910 was cultured in BHI supplemented with 0.6% sodium lactate (BHIL). These bacteria were grown anaerobically at 37 °C. *P. aeruginosa*PAO1 was cultured in Luria-broth (LB) (BD Difco™, USA), and MRSA ATCC BAA-2313™ was cultured in Nutrient Broth (NB) (BD Difco™, USA). These two bacteria were routinely grown aerobically at 37 °C. Culture of all strains was performed under BSL2 conditions and was approved by the Institutional Biosafety Committee. Because bacteria form multispecies biofilms on the bone that confer resistance to antimicrobials as compared to that of planktonic bacteria, for greater clinical relevance, multi-species bacterial biofilms were also employed. A previously characterized *ex vivo* clinical sample isolated from a peri-implant osteolytic lesion was cultured on porous hydroxyapatite discs to better simulate anti-biofilm efficacy under translational clinical conditions of bacterial bone infection. Isolation and culture conditions of the *ex vivo* ecological biofilm, which includes over thirty distinct bacterial taxa, have been previously described [16, 17].

**Table 1:** Bacterial species/strains used in this study

Strains	Characteristics	References
*V. parvula* PK1910	Wild-type	[21, 33]
MRSA ATCC BAA-2313™	Wild-type	[34]
*S. gordonii* DL1	Wild-type	[21, 35]
*P. aeruginosa* PAO1	Wound isolate	[36]

### Preparation of bioglass particles

Bioglass 45S5 and S53P4 were used in this study and were separated by particle size to determine particle size-dependent bacterial inhibitory effects as well as to mimic marketed product configurations for orthopedic bone graft applications. Bulk bioglass frit was placed in grinding jars with burundum grinding media and placed on a jar mill (U. S. Stoneware, East Palestine, OH) to pulverize the bioglass. The bioglass particulate was then sieved using stainless steel sieves on a mechanical shaker to separate it into the various size ranges (1 mm and 2 mm sieves for 1-2 mm particles; 500 µm and 710 µm sieves for 500–710 µm particles; 125 µm and 500 µm sieves for 90-710 µm particles; and 32 µm and 125 µm sieves for 32–125 µm particles.) Aliquots of particulate were packaged in heat-sealed pouches and sterilized using gamma irradiation (25 kGy).

### Antimicrobial effects of bioglass particles

All particles were weighed and mixed with different bacteria in corresponding broth. Based on previously published studies suggesting that concentrations up to 800 mg/ml of bioglass are required for complete growth inhibition of some bacterial species/strains [5], strain-specific pilot experiments were conducted to determine inhibitory concentrations with doses ranging from 50 mg/ml to 800 mg/ml. Overnight cultures of *S. gordonii*, *V. parvula*, *P. aeruginosa* and MRSA were centrifuged and resuspended with fresh corresponding media to an OD_600_ of 1.0. Next, resuspended bacterial cultures were diluted 1:1,000 into the media with pre-weighted bioglass particles. Bacterial cultures without particles were used as positive controls, and cultures with particles and without bacteria were used as sterility controls. The bacteria were incubated at 37 °C in aerobic (*P. aeruginosa* and MRSA) or anaerobic conditions (*S. gordonii* and *V. parvula*) as indicated for 24, 48 h or 72 h. Before measuring bacterial growth, bacteria were vortexed and suspended, and tubes were left standing until particles precipitated. Optical densities were measured at 600 nm, which directly corresponds to bacterial cell numbers in broth, to determine bacterial growth as compared to controls.

### Antibiofilm effects of bioglass

The efficacy of bioglass against established biofilms was assessed using the crystal violet staining method. For biofilm assays, MRSA and *V. parvula* were employed based on their ability to form single-species biofilms. Bacterial cells in log-phase were centrifuged and resuspended with fresh corresponding media to an OD_600_ of 1.0 for standardization. For biofilm formation, bacteria were inoculated at a 1:1,000 dilution in BHI broth supplemented with 0.6% sodium lactate (BHIL; *V. parvula*), or TSB broth with 0.5% Yeast Extract and 0.5% glucose (TSBYEG; MRSA). Then, 200 µl aliquots were added into sterile 96-well plates and incubated anaerobically (*V. parvula*) or aerobically (MRSA) at 37 °C for 24 h. Subsequently, to determine antibiofilm effects, Bioglass 45S5 and S53P4 with different particle sizes were weighed and aliquoted into the 96-well plates with the 24 h established biofilms at final concentrations of 100—200 mg/ml. After an additional 24 h incubation, the plates were washed with PBS three times to remove non-adhered cells, biofilm, and bioglass particles. Washed plates were then stained with crystal violet, dissolved with 30% acetic acid, and biomass measured at OD_562_ nm. Each assay was performed in triplicate wells and repeated three times. Biofilms stained with crystal violet without any stimulations served as controls.

### *Ex vivo* ecological biofilm formation and scanning electron microscopy (SEM)

To further study the antibiofilm effects of bioglass on multispecies biofilms, the multi-species *ex vivo* ecological biofilms described above were grown on porous HA-discs and SEM was used to assess the biofilm after treatment of bioglass 45S5/32–125 µm and S53P4/32–125 µm. Briefly, the working culture was prepared by inoculating frozen *ex vivo* biofilm stocks in TSB broth with 0.5% Yeast Extract and 0.5% sucrose (TSBYES) and grown anaerobically at 37 °C for 18 h. The overnight culture was diluted 1:10 into fresh TSBYES media and 1 mL bacterial culture was aliquoted into 24-well plate containing HA discs for ecological biofilm formation. To facilitate bacterial biofilm attachment, the sintered HA discs were modified with a round carbide burr at 2,000 rpm followed by acid etching with 37% orthophosphoric acid for 45 s, thus resulting in a microrough porous surface. The HA discs were sonicated in 70% ethanol prior to the experiment, and then placed into a 24-well plate and seeded with bacterial inoculums. The samples were cultured in an anaerobic jar at 37 °C for 24 h for the formation of ecological biofilms on HA-discs. After incubation, bioglass 45S5/32–125 µm and S53P4/32–125 µm were added into 24-well plate at the final concentration of 400 mg/mL, respectively. HA-disc without bioglass was used as control. The plate was cultured anaerobically at 37 °C for 24 h for bioglass treatment. After 24 h incubation, the HA-discs were washed with PBS times to remove non-adhered cells and biofilm and bioglass particles. The HA-discs were then fixed in a phosphate buffered 4% formaldehyde and 1% glutaraldehyde solution for 2 h, triple-washed in PBS for 3 min and post fixed in 1% osmium tetroxide for 1 h. After rinsing in Zetterquist’s buffer for 2 min, the HA-discs were then dehydrated and triple-washed with 70% and 95% ethanol for 15 min, followed by two 20 min rinses in 100% ethanol. The specimens were then treated in Hexamethyldisilazane (HMDS) for 5 min and air-dried in a desiccator. The samples were mounted on aluminum stubs, sputter-coated with gold palladium, and then examined with scanning electron microscopy.

### Fluorescence microscopy analysis

To validate the antibiofilm effect of bioglass, the *ex vivo* multi-species biofilms were grown on HA-discs as described above, and the mature biofilm was treated by 45S5 particles 32–125 µm, which were determined to have the best antibiofilm effects in the previous experiments and analyzed by fluorescence microscopy. Biofilms were prepared exactly as described above by inoculating HA-discs in 24-well plates. The plates were cultured in anaerobic jar at 37 °C for 24 h. After incubation, Bioglass 45S5/32–125 µm was added into 24-well plate at a final concentration of either 200 mg/mL or 400 mg/mL. The cultures without particles were used as control. The plate was cultured anaerobically at 37 °C for 24 h for bioglass treatment. After 24 h incubation, the 24-well plate was washed with PBS 3 times to remove unattached cells and bioglass particles. The biofilms were stained with Syto9 and Propidium Iodide (Live/Dead BacLight™ Bacterial Viability Kits) according to manufacturer’s instruction. After staining, biofilms were gently washed 3 times with PBS to remove dyes. Confocal imaging was performed using 40 × objective magnification on a KEYENCE BZ-X800 fluorescence microscope. 3D reconstruction was performed in ImageJ.

### Statistical analysis

To examine the differences between different bioglass compositions and particle sizes in treatment of the different bacterial strains, we performed statistical analyses via repeated-measures two-way ANOVA at different time points. Post-hoc testing using the False Discovery Rate (FDR) was used to determine differences in *pairwise comparisons for bacterial* inhibition related to particle type and concentration. A standard two-way ANOVA with post-hoc testing was used to examine variation in *V. parvula* and MRSA biofilm inhibition due to the particle type and particle size used. P-values were assessed as significant using FDR-adjusted alpha levels.

## Results

### Antibacterial effects of bioglass particles on planktonic bacteria

**Fig. 1:**
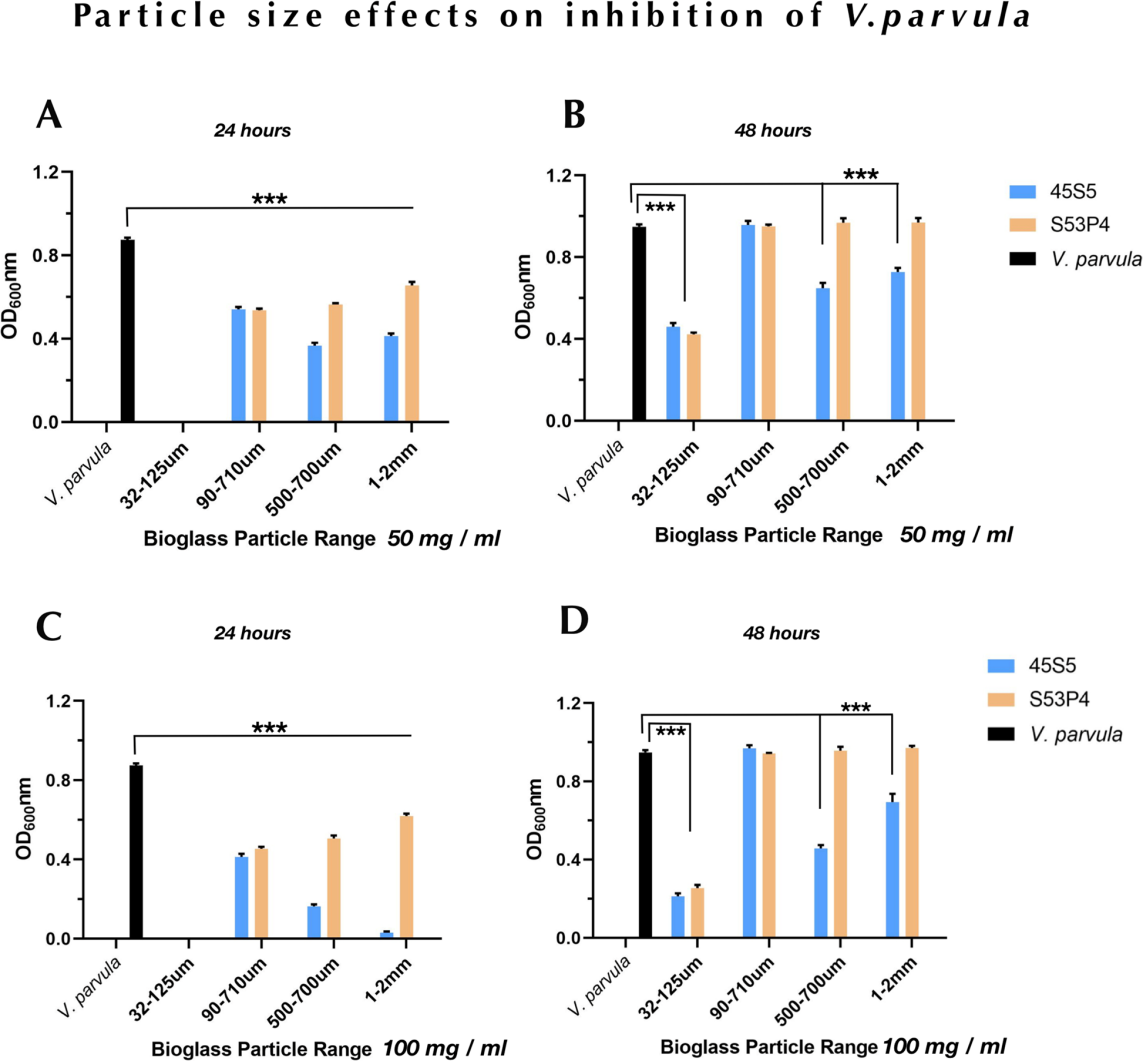
The inhibitory effects of bioglass particle size on *V. parvula* PK1910. 50 mg/ml (**A**, **B**) and 100 mg/ml (**C**, **D**) 45S5 and S53P4 were used to treat PK1910 for 24 h and 48 h. Data are representative of three experiments performed in triplicate. ^***^p < 0.001

To evaluate the antibacterial effects of bioglass particles, the bacterial cultures were treated with various size ranges of 45S5 and S53P4 bioglass. Bacterial growth was quantitated by measuring an optical density at 600 nm (OD_600_ nm). *Veillonella* are strictly anaerobic Gram-negative cocci and play crucial roles for oral biofilm formation and the *V. parvula* species are opportunistic pathogens [18–22]. Being that the *V. parvula* is causative to certain forms of osteomyelitis and is known to be resistant to multiple antibiomicrobials [10, 23], it was first assessed to determine composition- and particle-related antimicrobial effects of bioglass. Because *Veillonella* viability in liquid broth diminishes after 48 h, we measured OD_600_ nm after 24 h and 48 h incubation. As shown in Fig. 1, when 50 mg/ml (A&B) and 100 mg/ml (C&D) bioglass particles were incubated with *V. parvula* PK1910, the growth of this bacterium was completely inhibited by 45S5 and S53P4 with the size of 32–125 µm at 24 h time point (*p* < 0.001). However, the population partially recovered after 48 h of incubation, suggesting that *V. parvula* was not eradicated by either bioglass at the two concentration levels. In addition, after 48 h incubation, the 32–125 µm bioglass particles reduced bacterial growth as compared to the positive control (*p* < 0.001), while bacterial viability in the 90–710 µm particle range was comparable to controls (*p* > 0.05). Differential antimicrobial effects were noted when cultured for 48 h with 500–700 µm and 1–2 mm for both the 50 mg/ml and 100 mg/ml concentrations (Fig. 1B, D). Bacterial viability was fully recovered in these particle ranges in S53P4 groups, while 45S5 showed significantly greater inhibitory effects on the growth of *V. parvula* (*p* < 0.001) (Fig. 1). Because previous studies have suggested that bioglass antimicrobial effects are pH-mediated [7], we validated previous investigations by measuring the pH after 24 h culture and found that the maximal effect noted with the 32–125 µm range was consistent with the more alkaline conditions they produce as compared to larger particle groups (data not shown).

**Fig. 2:**
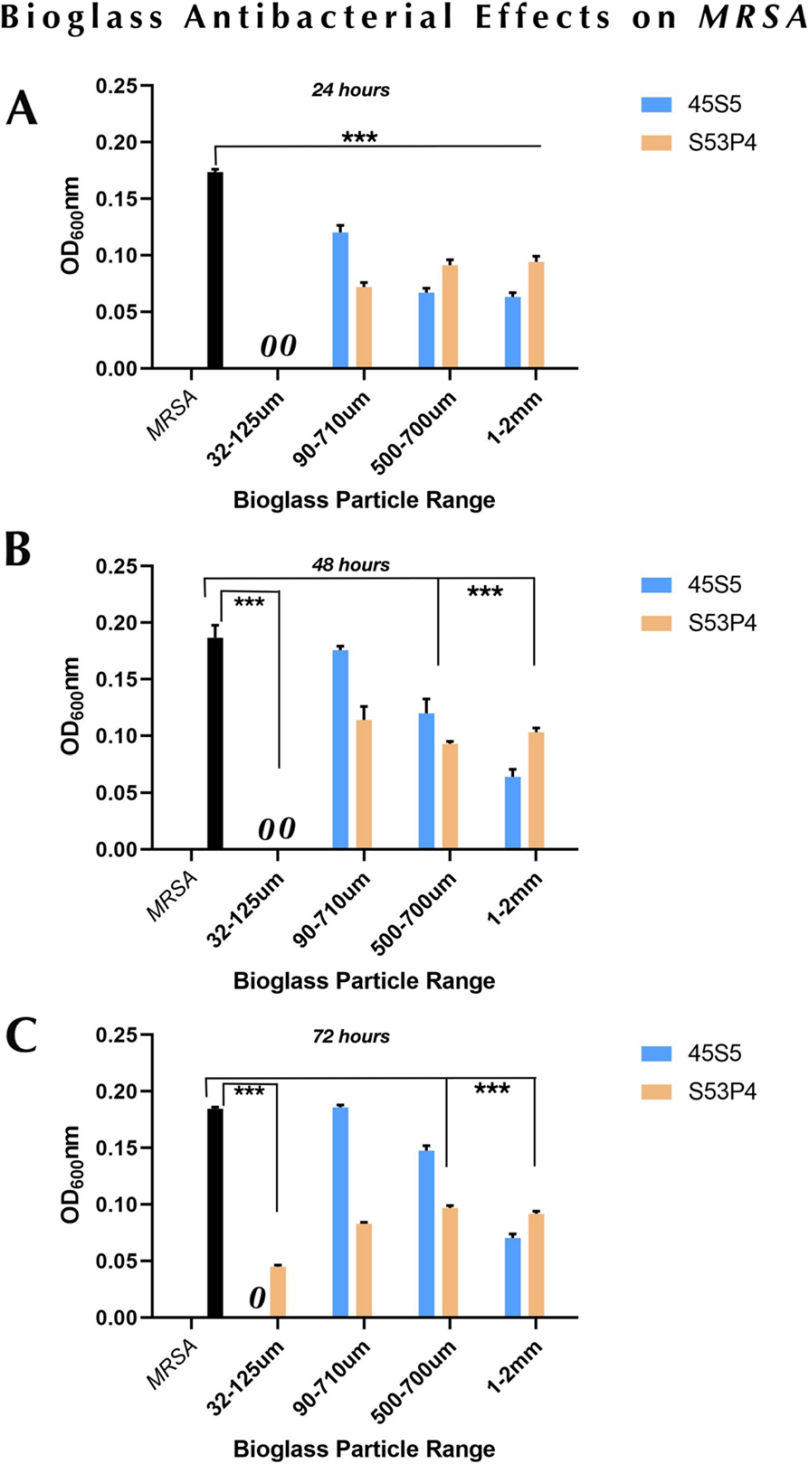
Antibacterial effects of bioglass 45S5 and S53P4 on MRSA. 200 mg/ml all size particles of 45S5 and S53P4 were used to challenge MRSA for 24 h (**A**), 48 h (**B**) and 72 h (**C**). Data are representative of three experiments performed in triplicate. ^***^p < 0.001

Due to its resistance to commonly used antibiotics, MRSA is notoriously more difficult to treat than most Gram-positive pathogens. Thus, we validated the findings of particle range effects on MRSA. In this study, we assessed the inhibitory effects of all sizes of bioglass particles on MRSA at a concentration of 200 mg/ml. Compared to non-treated bacterial cultures, all particles reduced MRSA growth at 24 h, and, notably, both 45S5 and S53P4 of 32–125 µm completely inhibit MRSA growth (Fig. 2A). After 48 h incubation, we did not observe any growth increase for the S53P4 bioglass group compared to 24 h cultures, however, MRSA growth was recovered in the group of 45S5/90–710 µm and 500–700 µm. Results were similar at 24 h, with no growth of MRSA measured for 45S5 and S53P4 particles of smallest size (Fig. 2B). Finally, we observed no difference for 45S5/1–2 mm and S53P4/90–710 µm, 500–700 µm and 1–2 mm after 72 h incubation. MRSA growth was recovered at levels similar to controls in the group of 45S5/90–710 µm and 500–700 µm. Interestingly, MRSA growth was slightly recovered in particle S53P4/32–125 µm, suggesting that MRSA could not be killed by this particle at 200 mg/ml concentration (Fig. 2C). The fact that MRSA was unable to grow in 45S5/32–125 µm suggests that this bioglass particle is the most effective at inhibiting MRSA.

**Fig. 3:**
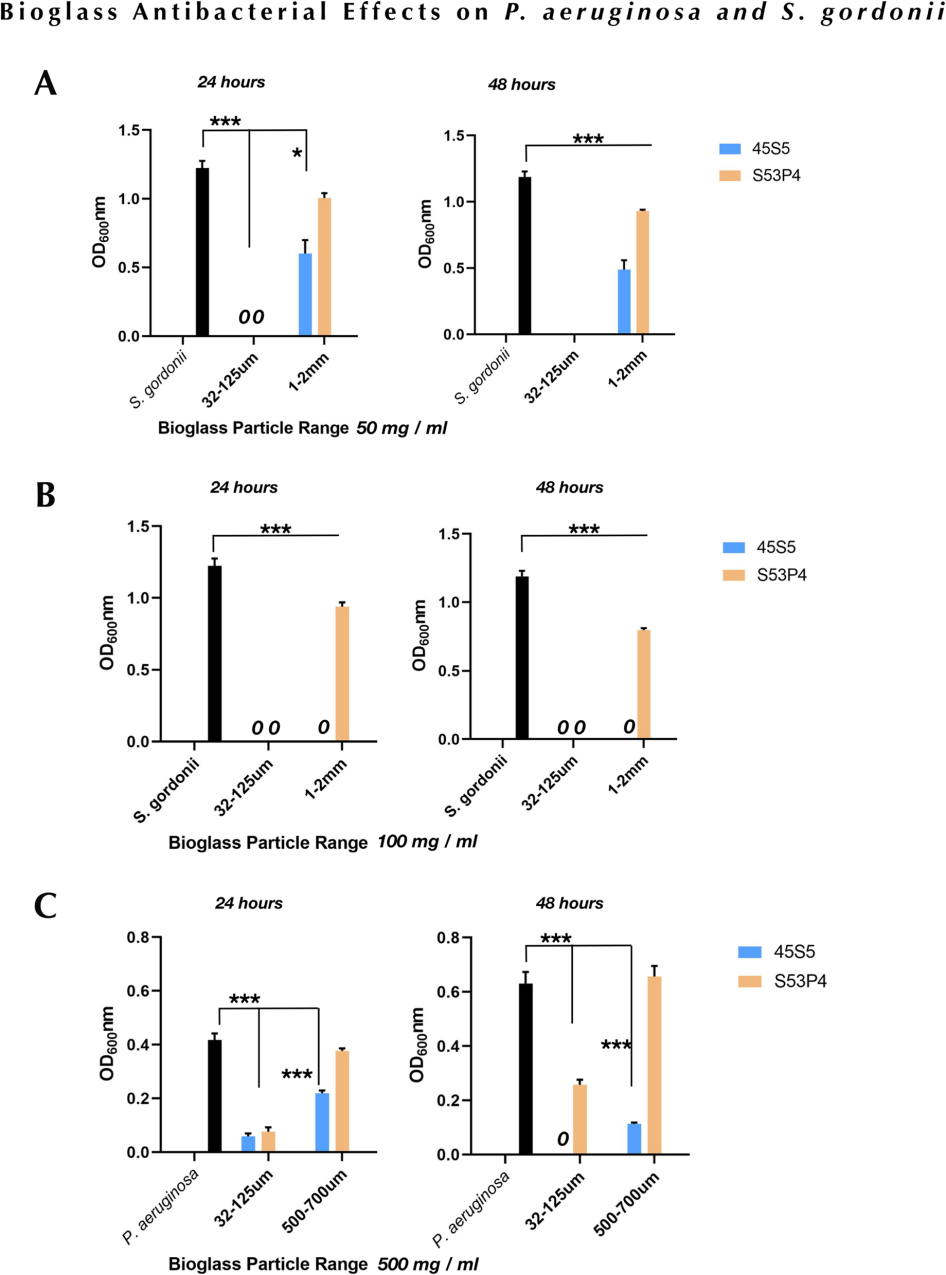
Antibacterial effects of bioactive glass 45S5 and S53P4 on *S. gordonii* and *P. aeruginosa*. 50 mg/ml (**A**) and 100 mg/ml (**B**) 45S5 and S53P4 with the size of 32–125 µm and 1–2 mm were utilized to treat *S. gordonii* for 24 h and 48 h. **C** *P. aeruginosa* was stimulated by 500 mg/ml 45S5 and S53P4 with the size of 32–125 µm and 500–700 µm for 24 h and 48 h. Data are representative of three experiments performed in triplicate. ^*^p < 0.05, ^***^p < 0.001

To further assess antibacterial effects of bioglass particles, *S. gordonii* and *P. aeruginosa* were employed in this study. The results of *Veillonella* and MRSA have demonstrated that both bioglass particles of the smallest size (32–125 µm) showed the most robust antimicrobial capability, so our studies for *S. gordonii* and *P. aeruginosa* focused on 45S5/32–125 µm and S53P4/32–125 µm. As shown in Fig. 3A, when 50 mg/ml bioglass particles were incubated with *S. gordonii* DL1, the growth of this strain was completely inhibited by 45S5 and S53P4 of the smallest size for 24 h and 48 h time points. Additionally, compared to bacterial control, particles 45S5 and S53P4 of the largest size (1–2 mm) can partially reduce DL1 growth after 24 h and 48 h incubation. As expected, when treated by 100 mg/ml bioglass particles, DL1 cannot grow in broth with 45S5/32–125 µm and S53P4/32–125 µm. Interestingly, 45S5/1–2 mm completely inhibited DL1 growth, and S53P4/1–2 mm showed slight inhibitory effect (Fig. 3B). As *P. aeruginosa* PAO1 is a clinical isolate, it may be more resistant to bioglass challenge. As expected, low concentrations of particles (100 mg/ml and 200 mg/ml) have no effect on PAO1 growth (data not shown). Thus, 500 mg/ml particles were used in this study (Fig. 3C). After 24 h incubation, 45S5/32–125 µm and S53P4/32–125 µm appear to impair PAO1 growth, and 45S5/500–700 and S53P4/500–700 µm showed slight inhibitory effect. Unexpectedly, PAO1 growth was further reduced in the mixture with 45S5 particles at 48 h, and we could not detect PAO1 growth in 45S5/32–125 µm, implying this bacterium might lyse after long-term incubation with 45S5 particles. Contrarily, PAO1 continued to grow in the presence of S53P4, and no difference was observed between bacterial control and S53P4/500–700 µm at 24 h and 48 h time points.

### Antibiofilm effects of bioglass particles on bacteria

**Fig. 4:**
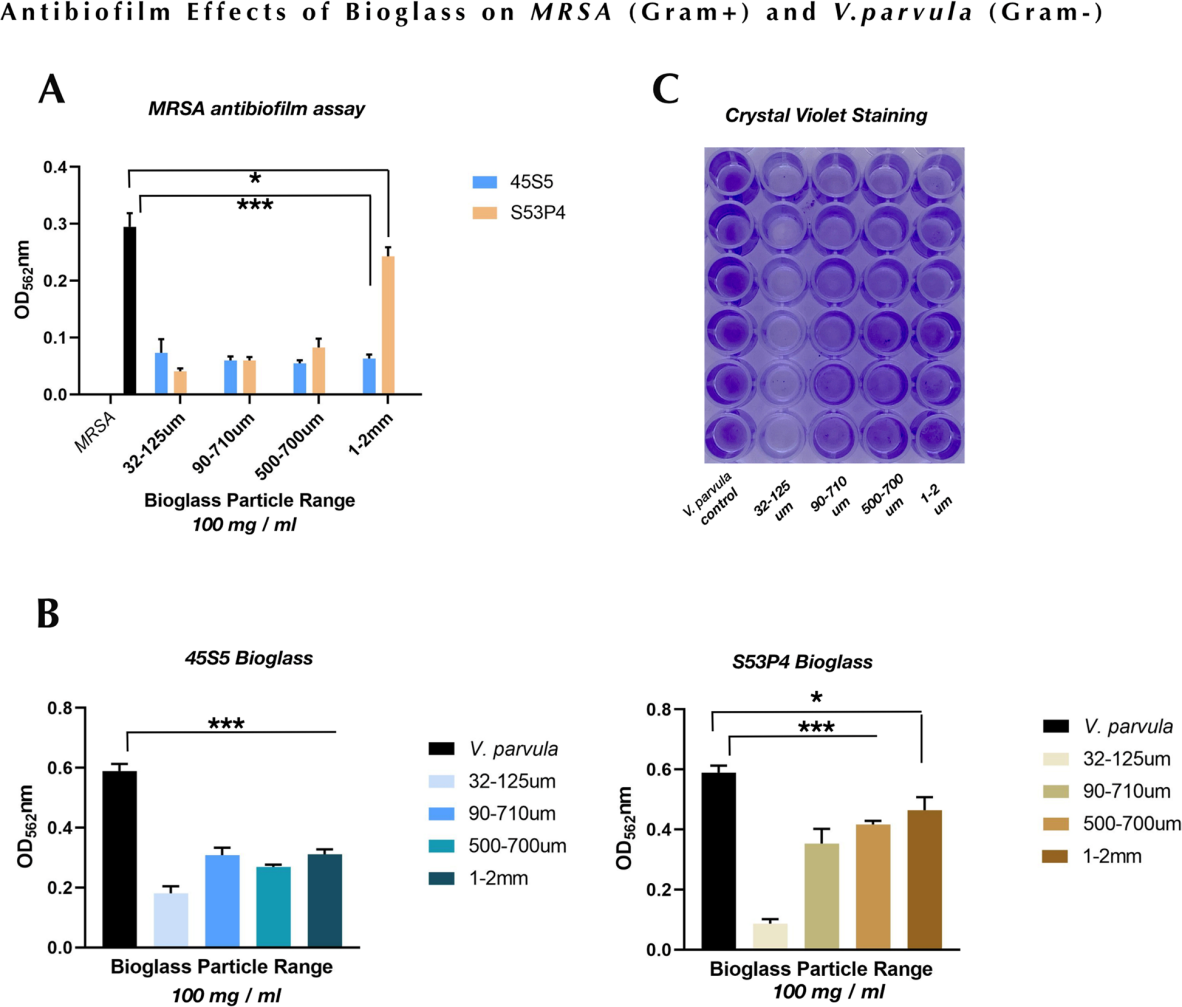
Antibiofilm effects of bioglass 45S5 and S53P4 on MRSA and *V. parvula*. **A** The established biofilm of MRSA was reduced by both types of bioglass at the concentration of 100 mg/ml. **B** The impaired effects of 100 mg/ml 45S5 and S53P4 on *V. parvula* biofilm. Data are representative of three experiments performed in triplicate. ^*^p > 0.05, ^***^p < 0.01. **C** Crystal violet staining shows *Veillonella* biofilm after inoculation with various ranges of S53P4

A biofilm is a group of microbial cells that are enclosed in a complex extracellular matrix. Microbes in biofilms are protected by the biofilm matrix, which confers resistance to various antimicrobial agents [24, 25]. To assess antibiofilm effects of bioglass particles, established biofilms of MRSA and *V. parvula* were treated with all size particles of 45S5 and S53P4. For the MRSA biofilm assay, the same concentration (200 mg/ml) used in antibacterial assays was used in this test. Interestingly, both 45S5 and S53P4 particles at 200 mg/ml completely impaired MRSA established biofilm (data not shown). Thus, we reduced particle concentrations to 100 mg/ml. As shown in Fig. 4A, after treatment with 100 mg/ml particles for 6 h, most bioglass particles except for S53P4/1–2 mm strongly reduced MRSA biofilm, and no obvious difference was observed among these bioglass particles. S53P4/1–2 mm slightly reduced MRSA biofilm compared to control.

To assess the antibiofilm activity of bioglass particles on *Veillonella* biofilm, 100 mg/ml bioglass particles were used to treat *V. parvula* PK1910 mature biofilm. As shown in Fig. 4B, all 45S5 particles can impair PK1910 biofilm, and 45S5/32–125 µm showed greater effect on antibiofilm compared to other size 45S5 particles. Similar to 45S5 group, all size S53P4 particles reduced PK1910 biofilm: S53P4/32–125 µm showed the strongest effect, and other particles slightly reduced mature PK1910 biofilm (Fig. 4B, C).

### Effects of bioglass particles on established biofilm of clinical sample on hydroxyapatite discs

**Fig. 5:**
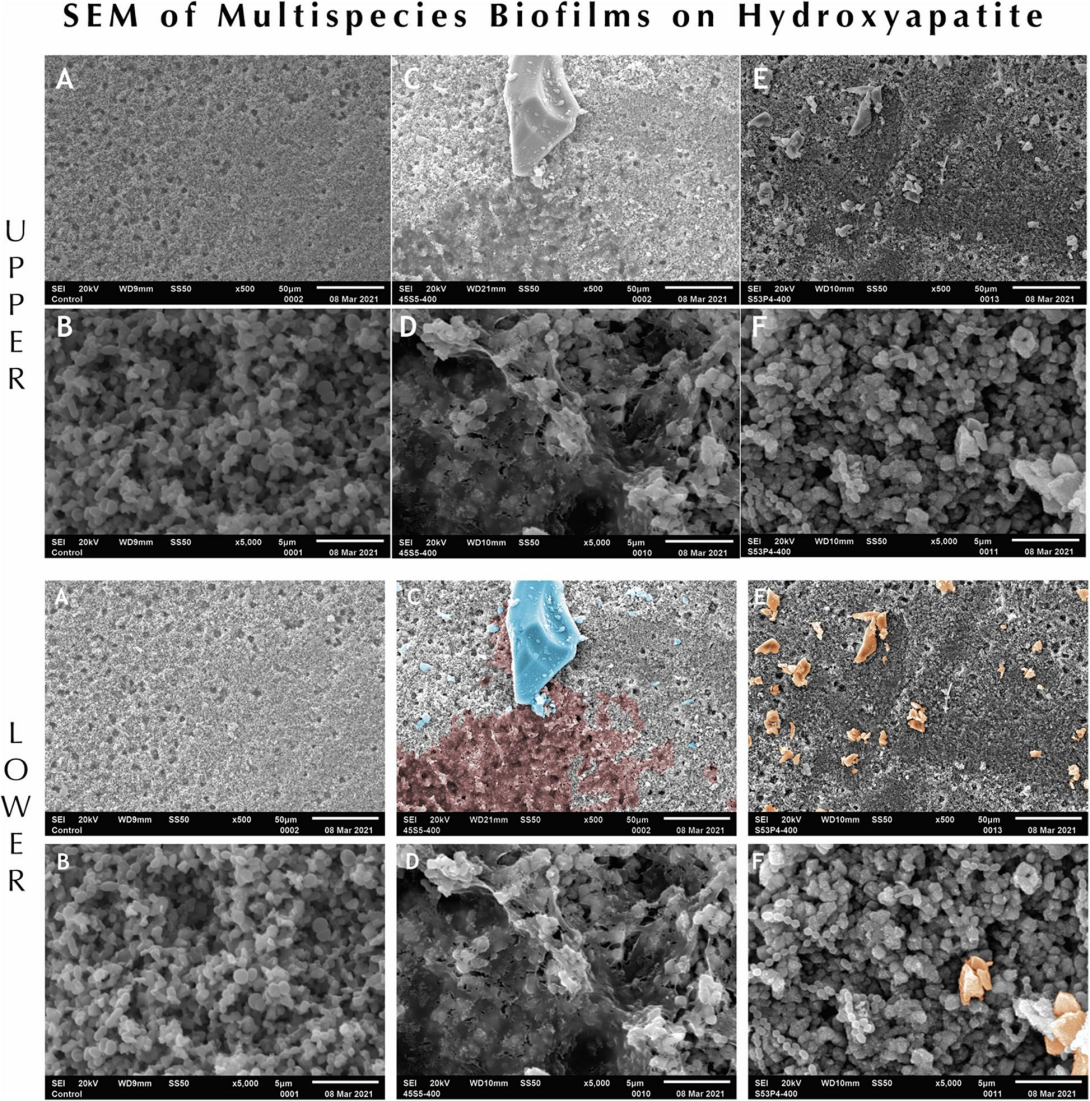
Scanning electron microscopy of *ex vivo* ecological biofilm formations of clinical samples. Upper panel: **A**, **B**: non-treated biofilm; **C**, **D**: particle 45S5/32–125 µm treated; **E**, **F**: particle S53P4/32–125 µm treated. Note the intact bacterial cells in the biofilm of S53P4/32–125 µm treated. Lower panel: Pseudocolor is used to depict the bioglass particles in each microphotograph for better identification

**Fig. 6:**
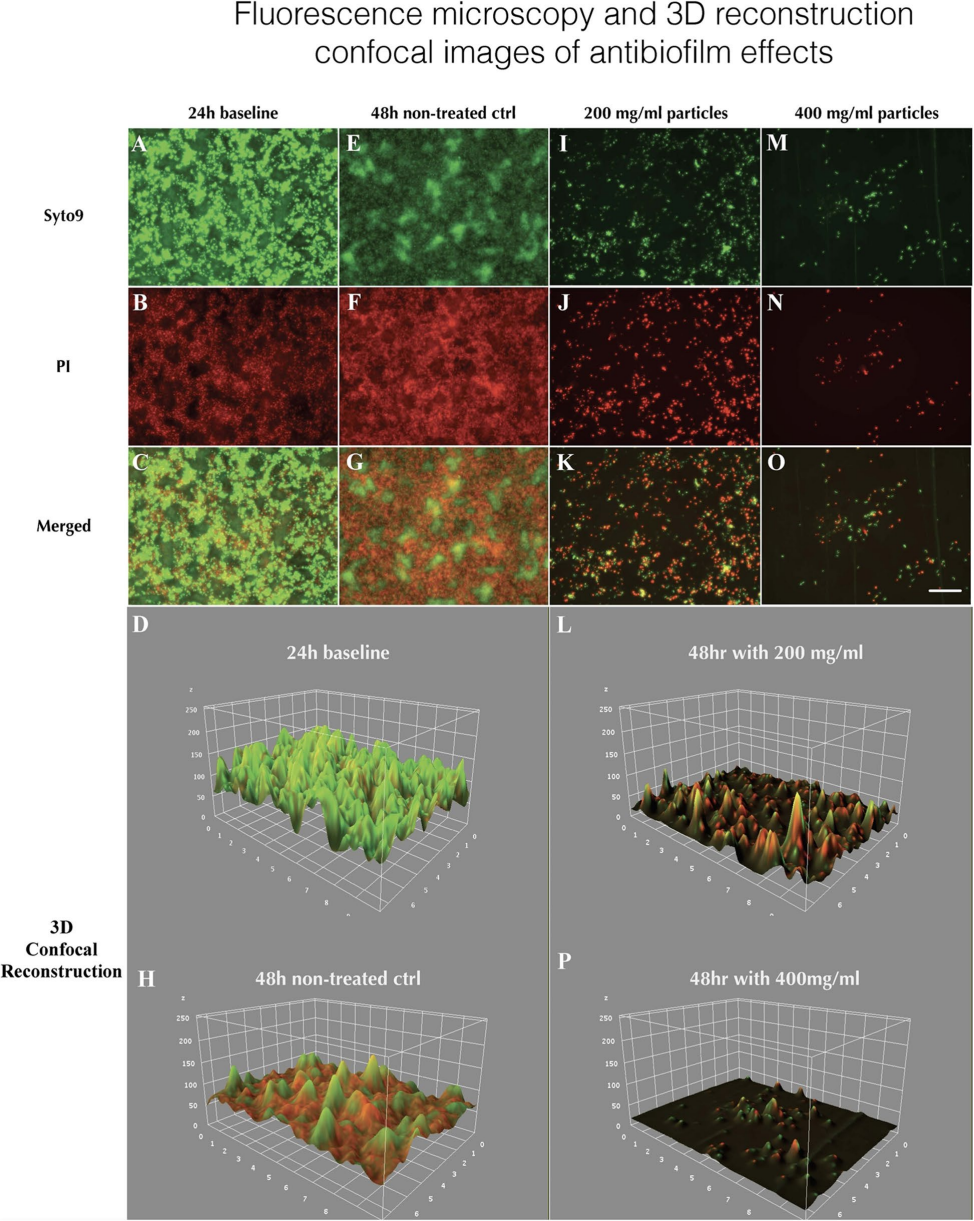
Fluorescence microscopy and 3D reconstruction images of *ex vivo* biofilm formations of clinical samples. Biofilms were stained with Syto9 (green) and PI (red), and fluorescence microscopy images were captured at 40 × magnification. Scale bar: 50 µm. **A**-**C** 24 h baseline biofilm; **E**–**G** 48 h non treated control; **I**-**K** *ex vivo* biofilm treated with 200 mg/ml 45S5/32–125 µm; **M**–**O** *ex vivo* biofilm treated with 400 mg/ml 45S5/32–125 µm. **D**, **H**, **L** and **P** 3D reconstruction images were generated based on corresponding merged images by using ImageJ

To further assess the antibiofilm effects of bioglass, clinical samples were used in this study to form a complex multi-species biofilm on hydroxyapatite (HA) discs. Due to our data demonstrating the smallest size bioglass showing the strongest activities of biofilm inhibition, SEM was used to observe and compare the biofilm population after treatment of 45S5/32–125 µm and S53P4/32–125 µm. As shown in Fig. 5, the *ex vivo* model employed could form strong biofilms on HA-disc surfaces (A & B); after treatment of bioglass, clinical biofilms were strongly reduced by 45S5/32–125 µm (C) and S53P4/32–125 µm (E), indicating both bioglass particles possess antibiofilm activities. The difference in biofilm height is indicated by differences in contrast in areas affected. It is interesting that live bacterial cells can be observed in the group of S53P4/32–125 µm treated biofilm (F); in contrast, compared to non-treated biofilm control (B) and S53P4/32–125 µm treated biofilm (F), most bacterial cells in 45S5/32–125 µm treated biofilm were dying or dead (D). Thus, having identified 45S5/32–125 µm as the group with the most potent antibiofilm efficacy, to further assess the impairing capability of this particle on established biofilm of clinical sample, two different concentrations (200 mg/ml and 400 mg/ml) of 45S5/32–125 µm were used to treat *ex vivo* ecological biofilms for live / dead imaging. Fluorescence microscopy was used to assess the surface coverage and viability of stained multi-species biofilms (Fig. 6). Multispecies biofilm samples exhibited the strongest fluorescence intensity after 24 h culture (A, B and C), demonstrating robust biofilm formation. As expected, treatment with bioglass 45S5/32–125 µm impaired and removed the established biofilms (I-K & M–O) after 24 h treatment showing significantly reduced surface coverage with both concentrations tested. Furthermore, 400 mg/ml treatment was more effective than 200 mg/ml (M–O vs I-K). The images of 3D reconstruction (D, H, L & P) derived respectively from corresponding merged fluorescent channels (C, G, K & O) indicated the biofilm spatial disruption and the elimination of the biofilm following treatment with minimal residual bacteria. These results demonstrated that bioglass particle 45S5/32–125 µm can both kill and remove robust clinical biofilms in a dose-dependent manner.

## Discussion

A series of independent and confirmatory experiments showed that regardless of other tested conditions, particle size was the key determinant of antibacterial activity; smaller particle diameters had greater effectiveness. Further, the role of the composition of the bioglass in antimicrobial efficacy varied among the tested strains, which responded differently to encounters with these antimicrobial particles. It has been reported that bioglass 45S5 and S53P4 have an antimicrobial effect against *Streptococcus mutans* [26, 27], in this study, another important *Streptococcus* species, *S. gordonii* showed the most overall growth inhibition compared to other tested bacteria, suggesting streptococci are sensitive to bioglass antibacterial effect. *S. gordonii* effects also seemed to be more composition dependent, whereas *V. parvula* levels were similar for both compositions. In the case of *S. gordonii*, growth inhibition was dependent on the type of particle used across most sizes and timepoints favoring the 45S5 parent composition. Similarities between the two particle types holds true across tested particle sizes and timepoints. The timepoint measured makes a significant difference, with a much stronger inhibitory effect at 24 h and bacterial growth nearly reaching control levels after 48 h for larger particle sizes. This suggests that even though the bacteria are very susceptible to the bioglass at 24 h, coping mechanisms may develop that provide tolerance to particles so bacteria can recover. An alternative explanation is that diameter related effects of bioglass, such as dissolution rate, ionic exchange ratios and pH-modulation, which all vary across particle ranges may have key roles in antimicrobial effects. Zhang et al. have demonstrated that bioglass inhibited the proliferation of *P. aeruginosa* at a concentration of 100 mg/ml [28], however, we found a clinical wound isolate *P. aeruginosa*strain PAO1 was able to survive at 100 mg/ml and 200 mg/ml bioglass concentrations, and partially inhibited at a high bioglass concentration (500 mg/ml). This is likely due to the higher tolerance of antibiotics and antimicrobials in clinical isolates. For both 45S5 and S53P4, the smallest size particles showed the strongest antibacterial effects for all tested bacteria. It has been reported that the bioactive glass can release ions from surface to increase osmotic pressure and pH in the environment, thus making the environment antagonistic to microbial growth [29]. So, the smaller particles have a greater surface area per unit mass, and then have increased potential to change environment.

Furthermore, the antibiofilm activity of bioglass S53P4 has been well studied. S53P4 showed the strong activity to reduce the biofilm produced by a broad range of microorganisms, such as *S. aureus, P. aeruginosa*, *K. pneumoniae*, *A. baumannii and S. epidermidis *[30–32]. In our study, 45S5 particles produced a stronger reduction in biofilm mass compared to S53P4 particles when considering small particle ranges, suggesting that this glass type has a greater potential for eradicating mature biofilm structures. Nonetheless, particle size remained the key determinant of effectiveness of these particles against biofilms as smaller particles reduced biofilms significantly more than larger particles across all experiments and consistent with planktonic assays. This is likely because smaller particles are closer in size to the bacteria, and thus are more apt to interact with and destroy bacterial biofilms. 32–125 µm particles of either type show much less remaining biofilm compared to each of the larger sizes, suggesting that this size of particle is most ideal for biofilm destruction. Interestingly, while the majority of previous published studies on antimicrobial effects of bioglass have focused on S53P4, the 32–125 µm 45S5 particles performed better at reducing biofilm biomass both against *V. parvula and S. gordonii* biofilms suggesting that the parent 45S5 may be more effective in eradicating infections caused by biofilms. In addition, the fact that the 32–125 µm 45S5 bioglass particles can kill and remove robust *ex vivo* clinical multi-species biofilms might help in deriving novel therapeutics for treatment and prevention of infectious diseases related to biofilm.

## Conclusion

In conclusion, there is ample evidence that suggests 45S5 has a greater degree of antibacterial activity when compared to S53P4. Therefore, the indications for 45S5 could be broadened to include the indications of S53P4 for clinical deployment in infected sites *in vivo.* Additionally, formulations of bioglasses could be optimized to contain mostly smaller size particles (32–125 µm) for improved infection treatment purposes. However, more studies should be conducted to consider if other types of particles and specifications could make a particle even more ideal for treatment of these infections and if these inhibitory effects are also seen in a clinical setting.

## Data Availability

The datasets generated and/or analyzed during this study are available from the corresponding author upon reasonable request.
